# Outpatient Psychotherapy Improves Symptoms and Reduces Health Care Costs in Regularly and Prematurely Terminated Therapies

**DOI:** 10.3389/fpsyg.2018.00748

**Published:** 2018-05-16

**Authors:** Uwe Altmann, Désirée Thielemann, Anna Zimmermann, Andrés Steffanowski, Ellen Bruckmeier, Irmgard Pfaffinger, Andrea Fembacher, Bernhard Strauß

**Affiliations:** ^1^Institute of Psychosocial Medicine and Psychotherapy, Universitätsklinikum Jena, Jena, Germany; ^2^Faculty of Applied Psychology, SRH University Heidelberg, Heidelberg, Germany; ^3^Psychotherapist in Private Practice, Feldafing, Germany; ^4^Psychotherapist in Private Practice, Munich, Germany; ^5^Bavarian Administration of Statutory Health Care Physicians, Munich, Germany

**Keywords:** outpatient psychotherapy, effectiveness, efficacy, cost reduction, premature termination

## Abstract

**Background:** In view of a shortage of health care costs, monetary aspects of psychotherapy become increasingly relevant. The present study examined the pre-post reduction of impairment and direct health care costs depending on therapy termination (regularly terminated, dropout with an unproblematic reason, and dropout with a quality-relevant reason) and the association of symptom and cost reduction.

**Methods:** In a naturalistic longitudinal study, we examined a disorder heterogeneous sample of *N* = 584 outpatients who were either treated with cognitive-behavioral, psychodynamic, or psychoanalytic therapy. Depression, anxiety, stress, and somatization were assessed with the Patient Health Questionnaire (PHQ). Annual amounts of inpatient costs, outpatient costs, medication costs, days of hospitalization, work disability days, utilization of psychotherapy, and utilization of pharmacotherapy 1 year before therapy and 1 year after therapy were provided by health care insurances. Symptom and cost reduction were analyzed using *t*-tests. Associations between symptom and cost reduction were examined using partial correlations and hierarchical linear models.

**Results:** Patients who terminated therapy regularly showed the largest symptom reduction (*d* = 0.981–1.22). Patients who dropped out due to an unproblematic reason and patients who terminated early due to a quality-relevant reason showed significant but small effects of symptom reductions (e.g., depression: *d* = 0.429 vs. *d* = 0.366). For patients with a regular end and those dropping out due to a quality-relevant reason, we observed a significant reduction of work disability (diff in % of pre-test value = 56.3 vs. 42.9%) and hospitalization days (52.8 vs. 35.0%). Annual inpatient costs decreased in the group with a regular therapy end (31.5%). Furthermore, reduction of symptoms on the one side and reduction of work disability days and psychotherapy utilization on the other side were significant correlated (*r* = 0.091–0.135).

**Conclusion:** Health care costs and symptoms were reduced in each of the three groups. The average symptom and cost reduction of patients with a quality-relevant dropout suggested that not each dropout might be seen as therapy failure.

## Introduction

Health care expenditures are large and are increasing steadily. In Germany, for example, 11.3% of the gross domestic product go into health care expenditures; these increased by 3.8% from 2015 to 2016 (Statistisches Bundesamt, 2018). Approximately 38.2% of the EU population suffers from a mental disorder (Wittchen et al., 2011). A proportion of 9.7% psycho-social impairments are one of most common reasons for work disability days (Wissenschaftliches Institut der Aok, 2017). Since mental disorders are widespread, monetary aspects of these disorders (e.g., work disability days) and their treatments become increasingly relevant (Wunsch et al., 2013; Castelnuovo et al., 2016).

There is a huge body of literature identifying mental disorders of particularly high prevalence among patients with unwarranted doctor visits and over-utilization of the health care system (Gabbard et al., 1997). Accordingly, an increase of medical service contacts, costs, hospitalization days, and work disability days during the 2 years before inpatient (Zielke, 1993) as well as outpatient psychotherapy (Kraft et al., 2004, 2006; Altmann et al., 2016). Especially depression and anxiety are associated with an increased use of general medical services (Candilis and Pollack, 1997; Simon and Katzelnick, 1997). Treatment and recovery of physical illness in patients suffering from mental problems are prolonged compared to patients not suffering from mental problems (Cohen et al., 1985; Levenson et al., 1990; Saravay and Lavin, 1994). In addition, mental disorders are commonly associated with long-term costs. Contrary to a decrease of sick leave in general, several German health insurance companies noticed an increase of sick leave caused by mental disorders by 30–50% (Bundespsychotherapeutenkammer, 2011).

The effectiveness of psychotherapy regarding psychological health has been repeatedly approved empirically (Lambert, 2013). Accordingly, psychotherapy has become an inherent part of health care services financed by statutory health insurance in many countries. In Germany, outpatient psychotherapy is of particular relevance, since 97.6% of the persons treated with a diagnosis of mental disorder between 2005 and 2007 were treated in outpatient therapy (Gaebel et al., 2011).

Besides the general effectiveness of outpatient psychotherapy, the change of health care costs and utilization in the context of psychotherapy has been investigated as a further aspect of treatment outcome. A meta-analysis of Chiles et al. (1999) demonstrated that 90% of English-language publications between 1967 and 1997 reported a cost reduction after outpatient or inpatient psychological interventions, and that 31% of studies found substantial economization above the cost-offset. On average, utilization is reduced by 15.7%, while the control group utilization increased by 12.3%. In the meta-analysis of Gabbard et al. (1997) all eight non-randomized studies and eight of the 10 studies with randomization found that psychotherapy reduces total costs. A meta-analysis of costs and benefits of long-term psychoanalytic therapy (de Maat et al., 2007) found reductions for the number of hospitalization days per year (between 59 and 85%), for the number of medical consultations per year (between 54 and 56%), and the number of medication users (between 19 and 70%). The review of Abbass and Katzman (2013) suggested that intensive short-term dynamic psychotherapy is cost-effective, for example, in terms of high return-to-work rates and reductions in hospital use. In the simulation study of Vasiliadis et al. (2016), epidemiologic and economic data from the literature were used. They found that every invested dollar in psychological services would lead to two dollars of savings for society.

However, in the meta-analysis of Chiles et al. (1999) only 18% of the studies considered patient samples with a mental disorder. The most studies examined psycho-educative interventions which were applied in the context of medical surgery. In addition, the meta-analysis of Gabbard et al. (1997) considered few individual psychotherapies. Most of them were psychological family interventions. Due to the need for cost analyses for outpatient psychotherapy of persons suffering from mental disorder (Chiles et al., 1999), a current study (Altmann et al., 2016) examined a disorder heterogeneous sample of 22,294 persons who were treated with outpatient therapy with cognitive behavioral therapy, psychodynamic therapy or psychoanalysis under naturalistic conditions. The authors found that direct health care costs were reduced by 10.1% on average of the costs in the year before outpatient psychotherapy, hospitalization days by 27.4%, and work disability days by 41.8%.

The findings that psychotherapy reduced symptom load and health care costs suggested that the change of symptom load is associated with the change of health costs. However, there is only weak empirical evidence for this assumption. Lazar et al. (2006) reported that despite the significant development of mental health, health care utilization is not significantly changed. Kraft et al. (2006) demonstrated that the change of psychological distress did not correlate significantly with the change of medical costs and change of hospitalization days. However, they found a significant correlation between the change of somatic distress and the change of medical costs and a marginal significant correlation with respect to the change of hospitalization days.

Considering all this evidence, our study examined associations between the change of psychological and somatic distress on the one side and health care costs such as costs for medication, work disability days, hospitalization days and utilization of psychotherapy and pharmacotherapy on the other side. In addition, we also tried to differentiate between regularly terminated therapies and premature terminations since premature terminations usually are understood as relevant for treatment quality. Studies about health cost changes in the context of premature terminations of outpatient psychotherapy are currently not available.

Besides the efficacy and effectiveness of psychotherapy, psychotherapy research increasingly investigates therapy failures (Strauss et al., 2012; Linden, 2013). It can be asked whether cost reduction also depends on the type of therapy termination (consensual termination vs. preliminary termination). Swift and Greenberg (2012) defined that a premature termination occurs if the patient unilaterally terminates the therapy against the therapist's recommendations. Furthermore, therapy has to be terminated before the problems are solved for which the therapy has been started. The meta-analysis of Swift and Greenberg (2014) revealed that—except depression and posttraumatic stress disorder (PTSD)—dropout rates did not differ between therapeutic approaches. For depression and PTSD, the lowest dropout rates were observed for integrative therapies. Strategies for therapists to avoid premature terminations are presented by Swift and Greenberg (2015). Evidence based strategies are, for example, strengthening of clients' feelings of hope, enhance their motivation to change, or maintain the therapeutic alliance.

It is important to note that not all dropouts are quality-relevant dropouts. Altmann et al. (2014b), Cinkaya et al. (2011), and Jacobi et al. (2011) differentiated between quality-relevant and unproblematic premature terminations. In the case of unproblematic dropouts, the patient is dropping out of therapy because he/she changed his/her residence or an improvement of the symptoms has already been achieved (Cinkaya et al., 2011; Jacobi et al., 2011). The percentage of dropout with quality-relevant reason in relation to all therapies ranges from 14.1% (Cinkaya et al., 2011) to 24.5% (Altmann et al., 2014b). Jacobi et al. estimated around 60% of all dropouts as are quality-relevant dropouts (Altmann et al.: 70.25%, Cinkaya et al.: 57.3%).

A recent meta-analysis (Swift and Greenberg, 2012) showed that the frequency of premature terminations is on average 21.9%. The discontinuation rate in pharmacotherapy is still 1.76 times higher than in psychotherapy (Swift et al., 2017). Possible adverse effects of premature terminations could be a lack of improvement of the level of mental functioning, resulting in negative consequences for the family, friends and colleagues as well as the frustration of the therapist (Swift and Greenberg, 2012). Many attempts are being made to identify predictors at the patient or therapist level that increase the probability of premature termination of psychotherapy. Predictors for a premature termination can be categorized in treatment based predictors, design based predictors, therapist characteristics and patient characteristics (Swift and Greenberg, 2012). In the context of the treatment-based predictors, a non-predefined duration of the intervention, a lack of manual-guided and university-based programs were found as predictors of an increased risk of discontinuation. On a design level, dropout definition, search strategy, and type of study (efficacy vs. effectiveness) seem to have an influence on the results. With regard to therapist characteristics, it was shown that 5.7% of the variance of dropout was explained by the therapist (Zimmermann et al., 2017). Therapist's experience seems to have an important impact on the probability of a premature termination (Swift and Greenberg, 2012). However, the existing body of research mostly investigated features on the patient level. Swift and Greenberg (2012) identified a low level of education as well as low age as significant predictors. In addition, the clinical diagnosis (Hamilton et al., 2011), therapists' experience, training and skills as well as the quality of therapeutic alliance (Roos and Werbart, 2013) have an influence. However, the findings with respect to the predictors are not consistent. Thus, socio-demographic as well as clinical data of a patient in the beginning of therapy do not appear to provide a great benefit in predicting the probability of termination. Identifying predictors after distinguishing between unproblematic dropouts and quality-relevant premature terminations may be useful.

In the present study, we wanted to consider matched cost- as well as questionnaire-data, and examined changes of direct costs before and after outpatient psychotherapy depending on type of therapy termination. According to Altmann et al. (2014b), Cinkaya et al. (2011), and Jacobi et al. (2011) we differentiated three groups: patients who regularly terminated treatment, dropouts with unproblematic reason (e.g., change of residence), and early terminators due to a quality-relevant reason (e.g., misfit of patient and therapist or when patient refused the indicated therapy). We tested the following hypotheses:

1. Patients who regularly terminated therapy show the highest reduction of symptoms from pre to post, the highest reduction of work disability days and hospitalization days as well as the highest reduction of direct health care costs regarding to the annual sum of 1 year before therapy vs. the annual sum of 1 year after therapy.
2. Quality-relevant dropouts show a slight decrease of symptoms, no change of work disability days, and an increase of health care costs because they visit other medical specialists to get another therapy due to their ongoing symptoms.
3. Independent of the kind of therapy termination, symptom reduction correlates with health care cost reduction in terms of higher symptom reduction should be related to higher cost reduction.

## Methods

### Background

The present study is based on the project “Quality Assurance in Ambulatory Psychotherapy in Bavaria” (QS-PSY-BAY) which examined the effect of outpatient psychotherapy on symptom reduction and cost reduction under naturalistic conditions (Strauss et al., 2015). Cost data and questionnaire data come from two separate data sources (for details see Strauss et al., 2015): About 79,000 individuals were randomly selected by health insurant funds using the database of all insured individuals of these funds treated with outpatient psychotherapy in the reference quarter. These completed no questionnaires. Only anonymized cost data were provided by the health insurant funds. *N* = 1,600 additional participants of the questionnaire study were recruited by their therapists. An inclusion criterion of the questionnaire study was that the patient was insured in one of the participating health insurance funds so that health care data could be tracked and provided. Previous studies focused on questionnaire data (Steffanowski et al., 2012; Altmann et al., 2014a,b, 2015; Strauss et al., 2015) or on cost data (Altmann et al., 2016). In the present study we analyzed for the first time merged dataset of questionnaire and cost data.

### Design

The presented study examined the outpatient psychotherapies from the project under naturalistic condition and has a quasi-experimental longitudinal design. We investigated the following three non-randomized groups: The *first group* consisted of patients who regularly terminated psychotherapy. The *second group* included patients who terminated their psychotherapy early due to an unproblematic reason such as for example hospitalization, change of residence, changes regarding to the partner, application of therapy extension was not approved by health insurance, or change of the health care insurance. Altmann et al. (2014b) showed that these reasons were not correlated with sub-optimal symptom reduction or therapeutic alliance. In other words, this type of early termination seemingly does not affect the quality of psychotherapy and seems to be mainly caused by an external reason. In contrast, therapies of the *third group* were early terminated due to a problematic and potentially quality-relevant reason. Such reasons comprised a misfit of patient and therapist, patient's refusal of an indicated therapy, discontinuation by patient, discontinuation by therapist, or consensual discontinuation of patient and therapist. Such reasons were associated with sub-optimal therapeutic alliance and low symptom reduction in a previous analysis (Altmann et al., 2014b).

In line with the German health care system, measurement time points for the questionnaires were the first (of five probatoric) therapy session, the end of the probatoric sessions (around session 5), before each extension of therapy (for cognitive-behavioral therapy (CBT) around the 25th session resp. for psychodynamic oriented therapy (PDT) around the 40th session), and at the end of therapy (for details see Steffanowski et al., 2012; Strauss et al., 2015). It should be noted that the first five therapy sessions are called “probatoric” sessions in which psychological problems of the patient are clarified and the corresponding mental disorder is diagnosed. Then, the financing of psychotherapy is requested toward the health care insurance of the patient. Depending on the severity of the mental disorder and the psychological approach, different contingents of therapy sessions are permitted (e.g., 25 sessions for CBT and short-term PDT, or 40 sessions for long-term PDT). At the end of therapy, an additional extension (an additional set of sessions) can be requested. Extensions were more commonly applied for patients with severe mental disorders, given a good therapeutic alliance and a sub-optimal therapy outcome at the time of extension (Altmann et al., 2014a).

### Patients and recruiting

**Figure 1:**
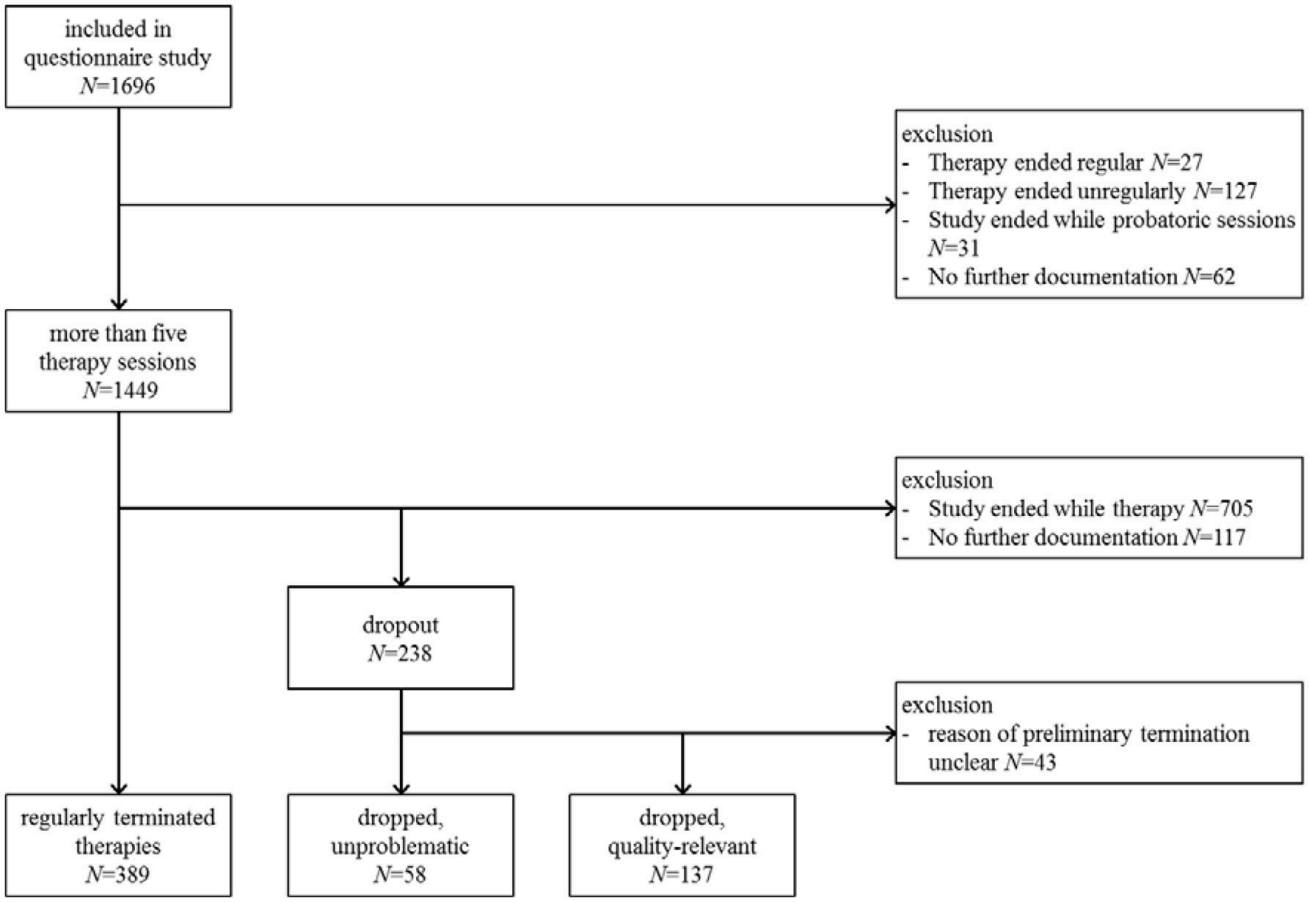
Flow chart.

In the questionnaire study of the QS-PSY-BAY project *N* = 1,692 patients were included (Steffanowski et al., 2012; Strauss et al., 2015). In Germany, health insurance funds reimburse costs for pre-defined sets of sessions depending on the therapeutic approach and severity of the mental disorder (e.g., short-term therapy 25 sessions; up to 300 sessions for psychoanalytic treatments). The first five therapy sessions (so called “probatory” sessions) focus on case history, diagnostic assessment and treatment planning. Based on these sessions a case report is written by therapist. Subsequently, psychotherapy (or an additional set of sessions) has to be requested from the health care insurance based on this report. In our sample, *N* = 1,449 patients started psychotherapy after the health care insurance accepted the patients' application for treatment (in German: “antragspflichtige Psychotherapie”). Out of these, *N* = 389 patients regularly terminated therapy, *N* = 58 patients terminated their psychotherapy early due to an unproblematic reason, and *N* = 137 patients terminated therapies due to a problematic quality-relevant reason within the study period. *N* = 865 patients were excluded from this study because the patients did not respond or the assessment interval ended while the therapy was still running so that therapy was not completed at the last measurement point (for details see Altmann et al., 2014b). Accordingly, the sample of this analysis includes *N* = 584 = 389 + 58 + 137 outpatient patients (see Figure 1). The minimum session number was five because we considered only requested therapies which included five probatory sessions.

All patients were recruited by their therapists. Participation was voluntary. To improve the recruiting rate, it was guaranteed and communicated to patients and therapist that (1) single patients will not be evaluated in terms of the individual time courses, only in an aggregated form, (2) single therapists will not be evaluated, and (3) psychotherapeutic approaches will not be compared. Patients were included from April 1st 2007 to June 30th 2009. The end of assessment for post therapy measures was the June 30th 2010 (Steffanowski et al., 2012). Patients who agreed to participate were included if they were insured by one of the participating health insurances, were over 18 years old, had a diagnosis of mental disorder (ICD10: F2–F6), were treated within individual therapy, and provided a written informed consent. Exclusion criteria, e.g., were a diagnosis of dementia (ICD10: F0) or addiction (ICD10: F1).

### Treatment

Patients (*N* = 584) were treated with individual outpatient psychotherapy either with cognitive behavior therapy (*N* = 282; 48.3%), psychodynamic therapy (*N* = 286; 49.0%), or psychoanalytic therapy (*N* = 16; 2.7%). Psychodynamic psychotherapy and psychoanalytic psychotherapy both are derived from psychoanalytic theory. Whereas, psychodynamic psychotherapy usually consists of a maximum of ~80 weekly sessions, psychoanalytic therapy usually lasts 240 sessions and more with a frequency of 2–3 sessions per week. Cognitive-behavioral therapy usually comprises 45–80 sessions. The duration of therapy ranged in this study from three to 177 weeks. Patients with regular therapy end (*M* = 57.7 weeks, *SD* = 25.5 respectively *M* = 28.3 sessions, *SD* = 22.0) stayed longer in psychotherapy than patients with quality-relevant early therapy termination (*M* = 56.0 weeks, *SD* = 29.0 respectively *M* = 13.4 sessions, *SD* = 20.6) and patients with unproblematic early therapy termination (*M* = 45.7 weeks, *SD* = 27.6 respectively *M* = 16.0 sessions, *SD* = 13.4).

The patients were treated by 120 therapists (1–20 patients per therapist, *M* = 4.9, *SD* = 4.0). 66 of the 120 therapists (55%) were female. The average age of therapists was 49.5 years (*SD* = 9.5). With regard to the *N* = 584 patients, 48.5% were treated by a female therapist. All 120 therapists were resident therapists and had a license to practice as a medical specialist (*N* = 31 specialists for psychosomatic medicine and psychotherapy, *N* = 9 specialists for psychiatry and psychotherapy, *N* = 12 general practitioners, *N* = 3 specialists for internal medicine) or a license as psychological psychotherapist (*N* = 65). University based outpatient clinics or comparable institutions did not participate in this study. On an average, the therapists had 9.4 years of professional experience (*SD* = 4.7) following the end of their professional training.

### Instruments

At the beginning of the outpatient psychotherapy the following socio-demographic data were assessed: age, gender, school qualification, status of permanent relationship, and employment status. Furthermore, we asked whether the patients already had a previous outpatient psychotherapy and/or previous inpatient psychotherapy. Additionally, we documented the primary diagnosis determined at the beginning of therapy.

In the present study, we only considered the subscales of the Patient Health Questionnaire (Gräfe et al., 2004) measuring depression, anxiety, stress, and somatization assessed at the beginning and end of therapy. We also computed the multiple status indicator (MSI) which reflects the average of the PHQ subscales depression, anxiety, stress, and somatization (cf. Steffanowski et al., 2012). Cronbachs α was good for depression (α = 0.84), anxiety (α = 0.87), somatization (α = 0.80) and MSI (α = 0.90), and acceptable for stress (α = 0.58; all coefficients based on baseline measures of *N* = 584 patients).

Direct health care costs were provided by the Bavarian Administration of Statutory Health Care Physicians (KVB) and six different health insurance companies of the “Verband der Ersatzkassen” (vdek). Single entries in our cost database were aggregated to quarter sums of inpatient costs, outpatient cost, and costs for medication. Due to the aggregation, we also aggregated over different reasons of costs. Therefore, costs could be caused by a wide range of diseases, e.g., influenca, cardiovascular diseases, or mental diseases etc. Likewise, a hospital stay could have been related to different treatments, for example, a treatment of a bone fracture, a psychosomatic treatment of chronic back pain, a psychiatric treatment of a mental disorder etc. Furthermore, we computed the 3-month sums of work disability days, hospitalization days, number of psychotherapy sessions and number of prescribed psychotropic drugs. It should be noted that the two latter are counted by the number of charge entries. As psychotropic drugs antipsychotics (ATC Code: N05A), anxiolytics (N05B), hypnotics and sedatives (N05C), as well as antidepressants (N06A) were counted. Quarter sums were matched with questionnaire data whereby with quarter sums being adjusted to the beginning and the end of the considered psychotherapy (for more details see Strauss et al., 2015; Altmann et al., 2016). This was done because the examined outpatient psychotherapies did not all start in the same quarter. The missing quarter sums were imputed (see below). Finally, we computed annual sums for 1 year before and 1 year after the considered psychotherapy. A value of 10 for work disability days before psychotherapy means that the health care insurance of this person had medical certificates for 10 days of sick leave registered in their database in the year before psychotherapy.

### Missing values

With regard to questionnaire data we had a complete dataset. In the QS-PSY-BAY project an electronic assessment system was developed and applied which ensured that a patient was contacted for an assessment at the right time and that all questionnaires were assessed completely, without any missing values (Steffanowski et al., 2012).

However, for patients with early terminated therapies the assessment of post therapy measures was not possible. Leaving their therapy prematurely, they also terminated the agreement of data assessment. Accordingly, we replaced the post therapy measure of these patients with the last available measure, which was the measure either obtained at the end of probatoric sessions (around the 5th session) or before an extension of therapy (for CBT around the 25th session resp. for PDT around the 40th session).

Missing data with respect to the cost data varied between 6.0 and 17.6%. With temporal distance of the reference quarter, the number of missing values increased. Missing values could be due to two reasons: individuals generated costs which were not documented or health-care costs which were not documented due to an individual's changed of the health insurance the individual's death (see Altmann et al., 2016). We tested if missing values were completely at random by using Little's MCAR test. The results indicate that missing values were completely at random (χ^2^ = 623.49, *df* = 720, *p* = 0.996). Missing cost data were imputed using a nonparametric approach by random forest imputation with the R package missForest (Stekhoven, 2011; Stekhoven and Bühlmann, 2012) with the following parameter settings: maximum number of iterations: 10, 1,000 random trees, and seed = 34. Predictors were socio-demographic variables, questionnaire and cost related data. It has been demonstrated that the missForest imputation leads to better results in comparison to the Hot Deck technique, mean imputation, or multivariate imputation (Misztal, 2013; Waljee et al., 2013). Due to the fact that cost data are not normally distributed and zero-inflated, we first transformed the cost data using logarithm transformation. MacNeil Vroomen et al. (2016) used a similar procedure in combination with a multiple imputation and showed most robust result in comparison to untransformed cost data. Fit-Indices of the imputation were good (NRMSE = 0.115, PFC = 0.000). After imputation, we re-transformed all cost data.

### Statistical analysis

Descriptive statistics of all three groups are first reported. We then compared the groups regarding pre-treatment variables. For categorical variables, we used χ^2^-test and Cramers V as effect size measure. Values between 0.1 ≤ *V* < 0.3 can be interpreted as small, 0.3 ≤ *V* < 0.5 as medium, and 0.5 ≤ V as large effect sizes (Cohen, 1988). Metric variables such as age were compared using ANOVA. Partial η^2^ is reported as effect size measure. Values between 0.01 ≤ η^2^ < 0.06 can be interpreted as small, 0.06 ≤ η^2^ < 0.15 as medium, and 0.15 ≤ η^2^ as large effect sizes.

In the next step, we estimated the change of symptoms from beginning to the end of therapy and the change of cost variables from 1 year before therapy to 1 year following therapy. As statistical test, we applied the Wilcoxon test because most variables were not normally distributed. The effect size was computed according to Borenstein et al. (2009) according to the formula: *d* = (M_post_-M_pre_)/s_within_ whereby s_within_ = s_diff_ /sqrt[2 ·(1-r_pre, post_)]. Values with a positive sign indicate a reduction of symptoms or health care costs, whereas a negative sign indicates an increase. The amount of d between 0.2 ≤ *d* < 0.5 can be interpreted as small, 0.5 ≤ *d* < 0.8 as medium, and 0.8 ≤ d as large effect size (Cohen, 1988).

Since raw data means do not take differences of sub-group characteristics (e.g., average session number) as well as nested data structure into the account, we applied a hierarchical linear model (HLM) with two random effects (“multiple patients per therapist” and “multiple therapists per therapeutic approach”). Dependent variables were PHQ scales assessed at the end of therapy and cost variables referring to annual costs in the year after therapy. Independent variables were gender, age, group (regularly terminated therapy, unproblematic dropout, quality-relevant dropout), number of therapy sessions, four scales of PHQ assessed at beginning of therapy (depression, anxiety, stress, somatization), cost and utilization variables referring to the year before therapy (inpatient, outpatient, and drug costs, work disability and hospital stay days, utilization of psychotherapy and pharmacotherapy in the year before therapy). The model included also interaction effects for the group and each other predictors (e.g., group × gender, group × age, etc.). Based on the estimated regression coefficients, adjusted outcome averages were estimated. These group averages refer to the characteristics of initial symptoms and annual costs of dropouts with problematic reason (see **Table 2**). This method was, for example, also applied by Holtforth et al. (2011) and Altmann et al. (2014a). A detailed description of the mathematical foundation of the estimation of adjusted averages and average effects in quasi-experimental settings is described by Mayer et al. (2016).

Next, we related cost savings and expenditures depending on the kind of therapy termination. Cost savings were operationalized as sum of annual cost in the year before the therapy (inpatient, outpatient, and drug costs) minus the sum of annual costs in the year after therapy plus savings for work disability days. Based on the statistics of the Bundesanstalt für Arbeitsschutz und Arbeitsmedizin (2016), one work disability day is on average related to 270€ operating company expenses (sum of 105€ production loss and 165€ loss of gross value added). Regarding expenditures of outpatient psychotherapy, we assumed that the first five probatory sessions counted with 45€ and every further session with 90€ in concordance with current regulation on fees in Germany. It should be noted that simultaneous pharmacotherapy was not documented so that the expenditures are possibly underestimated.

To finally quantify the association between change of symptoms and change of direct health care costs, we computed difference variables based on pre-post-symptom measures as well as on pre-post-cost-measures. According to Kraft et al. (2006), we applied partial correlations of the difference variables controlling for age and gender. A positive sign of the correlation coefficient indicates that a reduction of symptoms corresponds with a reduction of health care costs. A negative sign means that, while symptoms were reduced, health care costs increased or vice versa. The amount of r between 0.1 ≤ *r* < 0.3 can interpreted as low, 0.3 ≤ *r* < 0.5 as moderate, and 0.5 ≤ r as high (Cohen, 1988).

Furthermore, we applied hierarchical linear models to examine the association between change of symptoms and change of direct health care costs taking covariates and nested data structure into account. We used change of direct costs (annual sum of the year after current outpatient psychotherapy minus annual sum of the year before current therapy) as dependent variables. Covariates were patient's gender and age, inpatient costs, outpatient costs, drug costs, work disability days, hospital stay days in the year before, number of sessions of the current psychotherapy, baseline measure of MSI, and change of MSI (post minus baseline measure). We applied a random intercept model with two random effects: multiple patients per therapist and multiple therapists per therapeutic approach. As effect size measures standardized regression coefficients were reported.

For all statistical analysis, we used SPSS version 21. The significance level was α = 0.05 for all tests. Due to the explorative character of our study we did not apply an adjustment of *p*-values due to multiple testing.

## Results

### Sample description

Table 1 summarizes descriptive statistics of the three groups with regular termination (*N* = 389), dropouts with unproblematic reason (*N* = 58), and dropouts with quality-relevant reason (*N* = 137). Univariate tests indicated that the groups differed significantly with regard to gender, age, school qualification, relationship status and working status (see Table 1). All other variables were not significant. The group of patients who terminated their therapy regularly had the highest proportion of male patients, the highest mean age, and the highest proportion of employed patients. Compared to the other groups, patients who terminated their therapy due to a quality-relevant reason most often had no high school graduation, no permanent or shifting relationships. Furthermore, they most often were unemployed. Dropouts with unproblematic reason most often had a high school graduation and a long-term relationship with supporting partners.

**Table 1:** Characteristics of considered sub-samples.

	**Regularly terminated (*N* = 389)**	**Dropout with unproblematic reason (*N* = 58)**	**Dropout with quality-relevant reason (*N* = 137)**	**Statistics of group comparisons**
	**M (SD)/N in %**	**M (SD)/N in %**	**M (SD)/N in %**	
Female patients	73.8%	84.5%	83.9%	χ^2^ = 7.878, *df* = 2, *p* = 0.019, *V* = 0.116
Age of patients (in years)	*M* = 40.9 (12.8)	*M* = 33.1 (10.6)	*M* = 37.9 (12.6)	*F* = 11.218, *df_1_* = 2, *df_2_* = 581, *p* < 0.001, η^2^ = 0.037
	**School-leaving qualification**		
No high school graduation	66.1%	48.3%	71.5%	χ^2^ = 9.886, *df* = 2, *p* = 0.007, *V* = 0.130
High school graduation	33.9%	51.7%	28.5%	
	**Permanent relationship**		
Yes, partner gives support	51.2%	58.6%	40.2%	χ^2^ = 17.967, *df* = 6, *p* = 0.006, *V* = 0.124
Yes, but partner gives no support	14.1%	17.2%	11.7%	
No permanent relationship	29.6%	17.2%	35.0%	
Shifting relationships	5.1%	6.9%	13.1%	
	**Working status**		
Employed	63.0%	56.9%	48.2%	χ^2^ = 25.738, *df* = 8, *p* = 0.001, *V* = 0.210
Unemployed	5.4%	6.9%	16.8%	
Retired	6.9%	1.7%	5.1%	
Housewife	8.5%	8.6%	10.2%	
Other	16.2%	25.9%	19.7%	
	**Had an early psychotherapy**		
Outpatient	27.0%	24.1%	35.8%	χ^2^ = 4.48, *df* = 2, *p* = 0.106, *V* = 0.088
Inpatient	16.5%	17.2%	24.1%	χ^2^ = 3.997, *df* = 2, *p* = 0.136, *V* = 0.083
	**Primary diagnosis (according ICD10)**		
Mild depression (F32.0, F33.0, F34.1)	13.9%	13.8%	14.6%	χ^2^ = 21.79, *df* = 16, *p* = 0.15, *V* = 0.137
Moderate depression (F32.1, F33.1)	27.8%	24.1%	24.8%	
Severe depression (F32.2/3, F33.2/3)	8.7%	6.9%	9.5%	
Phobic anxiety disorders (F40)	8.2%	1.7%	3.7%	
Other anxiety disorders (F41)	11.8%	5.2%	13.9%	
Adjustment disorders (F43.2)	8.0%	15.5%	11.0%	
Somatoform disorders (F45)	5.4%	3.5%	2.2%	
Personality disorder (F6)	2.8%	5.2%	5.8%	
Other	13.4%	24.1%	14.6%	

*M, mean; SD, standard deviation; χ^2^, Chi-square statistics; df, degree of freedom; p, p-value; V, Cramers V an effect size measure; F, F statistics; η^2^, Eta-square an effect size measure*.

Additionally, we examined the association between group and symptom load at the beginning of therapy. We found a significant difference with regard to depression (*F* = 3.391, *df*_1_ = 2, *df*_2_ = 581, *p* = 0.034, η^2^ = 0.012). Patients with quality-relevant premature termination showed on average more depressive symptoms than patients who regularly terminated the therapy (diff = 0.16, *SE* = 0.063, *p* = 0.011). Significant differences in the health costs of the year before therapy were not found (no significant overall-test). However, a marginal significant effect was found with respect to the tests for inpatient costs (*F* = 2.814, *df*_1_ = 2, *df*_2_ = 581, *p* = 0.061, η^2^ = 0.010) and number of hospitalization days (*F* = 2.565, *df*_1_ = 2, *df*_2_ = 581, *p* = 0.078, η^2^ = 0.009).

### Symptom load and costs before and after outpatient psychotherapy

Statistics related to the symptom reduction from pre to post and the reduction of direct health care costs from 1 year before to 1 year after outpatient psychotherapy are listed in Table 2 depending on the subgroup. With regard to symptom reduction, we observed large effects for patients who regularly terminated their psychotherapy. In contrast, only small effects were found regarding patients with early termination. Significant changes of health care costs were observed for patients with regularly terminated therapies. The annual amount of inpatient costs was reduced by 331.72€ (diff in % of pre-test = 31.5%), concurrently, the annual amount of outpatient costs was increased by 84.86€ (diff in % = −13.2%). The largest amount of reduction of inpatient costs was found for dropouts with unproblematic reason (diff = 1096.90€, diff in % = 46.2%). However, the change was not significant (*p* = 0.392) in this small group (*N* = 58). Significant decreases of work disability days and hospitalization days were found for patients with regularly terminated therapies (diff = 12.9, diff in % = 56.3%, *p* < 0.001 days resp. diff = 2.55 days, diff in % = 52.8%, *p* = 0.001) and—against our expectation—also for dropouts with quality-relevant reason (diff = 21.69 days, diff in % = 42.9%, *p* = 0.026 resp. diff = 2.46, diff in % = 35.0%, *p* = 0.032). The largest amount of decrease of hospitalization days was observed for patients with early, though unproblematic termination, but the change was not significant (diff = 6.2 days, diff in % = 54.8%, *p* = 0.763) for this small group (*N* = 58). At last, the number of psychotherapies in the year after the considered psychotherapy increased significantly in all three groups.

**Table 2:** Symptom load and direct health care costs before and after an outpatient psychotherapy (costs reported in € and annual sums).

	**M1**	**SD1**	**M2**	**SD2**	**r(pre,post)**	***d***	***p***
**DEPRESSION (PHQ)**							
Regular terminated	1.32	(0.61)	0.63	(0.51)	0.380	1.220	<0.001
Dropped, unproblematic	1.32	(0.71)	1.07	(0.59)	0.618	0.366	0.002
Dropped, quality-relevant	1.48	(0.66)	1.20	(0.64)	0.648	0.429	<0.001
**ANXIETY (PHQ)**							
Regular terminated	1.44	(0.72)	0.59	(0.57)	0.290	0.981	<0.001
Dropped, unproblematic	1.43	(0.91)	1.21	(0.74)	0.581	0.187	0.047
Dropped, quality-relevant	1.59	(0.72)	1.27	(0.74)	0.661	0.332	0.000
**STRESS (PHQ)**							
Regular terminated	0.85	(0.38)	0.49	(0.35)	0.483	0.998	<0.001
Dropped, unproblematic	0.85	(0.36)	0.75	(0.40)	0.636	0.269	0.021
Dropped, quality-relevant	0.92	(0.36)	0.80	(0.35)	0.620	0.364	<0.001
**SOMATIZATION (PHQ)**							
Regular terminated	0.78	(0.36)	0.50	(0.33)	0.495	1.094	<0.001
Dropped, unproblematic	0.71	(0.40)	0.66	(0.34)	0.716	0.208	0.056
Dropped, quality-relevant	0.83	(0.38)	0.72	(0.37)	0.749	0.371	<0.001
**INPATIENT COSTS**							
Regular terminated	1051.80	(3199.86)	720.08	(3099.51)	0.066	0.105	0.005
Dropped, unproblematic	2373.03	(6924.72)	1276.13	(3631.52)	0.231	0.193	0.392
Dropped, quality-relevant	1318.24	(4276.43)	874.15	(3509.51)	−0.018	0.114	0.120
**OUTPATIENT COSTS**							
Regular terminated	640.92	(996.45)	725.78	(760.36)	0.215	−0.095	0.004
Dropped, unproblematic	517.05	(516.07)	524.93	(684.84)	0.237	−0.013	0.051
Dropped, quality-relevant	498.76	(411.91)	872.67	(1419.25)	0.163	−0.343	0.678
**DRUG COSTS**							
Regular terminated	455.77	(1837.38)	398.33	(1322.01)	0.691	0.034	0.312
Dropped, unproblematic	366.09	(871.00)	283.38	(678.30)	0.411	0.105	0.189
Dropped, quality-relevant	295.52	(809.99)	641.83	(3992.82)	0.168	−0.113	0.435
**WORK DISABILITY DAYS**							
Regular terminated	22.93	(54.28)	10.03	(26.27)	0.264	0.292	<0.001
Dropped, unproblematic	14.13	(30.03)	13.91	(45.48)	0.071	0.006	0.152
Dropped, quality-relevant	21.69	(48.75)	12.39	(37.98)	0.248	0.212	0.026
**HOSPITAL STAY**							
Regular terminated	4.83	(18.96)	2.28	(10.68)	0.094	0.164	0.001
Dropped, unproblematic	11.32	(30.63)	5.12	(17.74)	0.050	0.247	0.763
Dropped, quality-relevant	7.03	(22.76)	4.57	(21.37)	0.049	0.112	0.032
**PSYCHOTHERAPY (NUMBER OF GOP)**							
Regular terminated	0.11	(0.47)	0.31	(0.88)	0.068	−0.276	<0.001
Dropped, unproblematic	0.09	(0.27)	0.48	(1.12)	0.179	−0.448	0.001
Dropped, quality-relevant	0.17	(0.69)	0.45	(1.15)	0.144	−0.289	0.011
**PHARMACOTHERAPY (NUMBER OF GOP)**							
Regular terminated	0.51	(0.99)	0.49	(1.05)	0.557	0.016	0.112
Dropped, unproblematic	0.49	(0.90)	0.39	(0.90)	0.718	0.105	0.375
Dropped, quality-relevant	0.55	(1.07)	0.61	(1.15)	0.474	−0.060	0.836

*M1, mean pre; M2, mean post; SD1, standard deviation pre; SD2, standard deviation post; r, Spearman correlations; raw data means without adjustment for differences of sample characteristics*.

**Figure 2:**
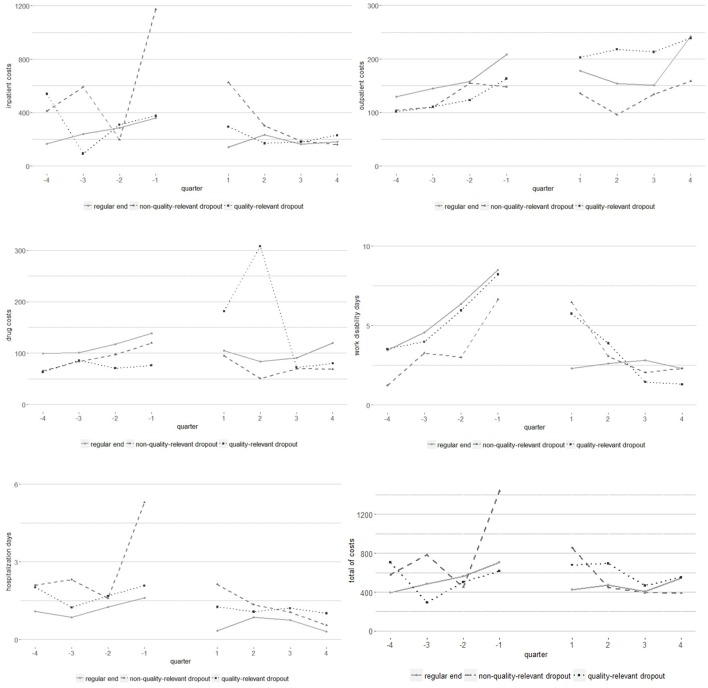
Time course of direct healt care costs (quater sums before and after outpatient psychotherapy).

To give a better impression of the change of medical costs, quarter averages are shown in Figure 2 depending on the kind of therapy termination.

The averages in Table 2 refer to sub-samples with different characteristics. The average session number of regularly terminated therapies was, for example, 28.3 sessions whereas dropouts with quality-relevant reason were treated 13.4 sessions on average. Accordingly, we applied a HLM and estimated adjusted outcome averages based on regression coefficients of the HLM. The adjusted averages reported in Table 3 refer to characteristics of dropouts with quality-relevant reasons. The analyses revealed that unproblematic dropouts and quality-relevant dropout had a similar symptom load at the end of therapy given a treatment with an equal session number of 13.4 sessions. For cost variables, work disability days and utilization of pharmacotherapy in the year after therapy, we found no group differences. On a descriptive level, it can be stated that unproblematic dropouts had the highest inpatient cost and work disability days in the year after therapy. Quality-relevant dropouts had the highest medication costs, outpatient costs and utilization of pharmacotherapy in the year after psychotherapy. Significant mean differences were found for utilization of psychotherapy in the year after therapy. The highest average was observed for unproblematic dropouts and the lowest for patients with regularly terminated therapy.

**Table 3:** Adjusted averages of symptoms at end of therapy and costs in the year after psychotherapy (in €) and statistics of overall test comparing a three group averages.

	**Regular terminated**		**Dropped, unproblematic**		**Dropped, quality-relevant**		**Comparison**
	***M***	***(SD)***	***M***	***(SD)***	***M***	***(SD)***	
Depression (PHQ)	0.65	(0.63)	1.08	(0.67)	1.20	(0.48)	*df_1_* = 2, *df_2_* = 526.3, *F* = 60.7, *p <* 0.001
Anxiety (PHQ)	0.59	(0.75)	1.16	(0.77)	1.28	(0.56)	*df_1_* = 2, *df_2_* = 533.4, *F* = 73.4, *p <* 0.001
Stress (PHQ)	0.49	(0.41)	0.78	(0.42)	0.80	(0.30)	*df_1_* = 2, *df_2_* = 531.8, *F* = 52, *p <* 0.001
Somatization (PHQ)	0.50	(0.39)	0.72	(0.38)	0.72	(0.28)	*df_1_* = 2, *df_2_* = 535.9, *F* = 33.3, *p <* 0.001
Inpatient costs	855.96	(4724.03)	1571.13	(4649.25)	961.12	(3405.79)	*df_1_* = 2, *df_2_* = 535.4, *F* = 0.6, *p* = 0.538
Outpatient costs	916.25	(5913.61)	906.79	(2561.57)	1077.18	(3495.23)	*df_1_* = 2, *df_2_* = 533.9, *F* = 1.3, *p* = 0.265
Drug costs	355.15	(2693.84)	288.07	(2905.28)	639.74	(2060.14)	*df_1_* = 2, *df_2_* = 539, *F* = 0.9, *p* = 0.402
Work disability days	11.35	(46.47)	22.14	(44.27)	12.55	(33.07)	*df_1_* = 2, *df_2_* = 516.8, *F* = 1.6, *p* = 0.209
Hospital stay days	2.92	(25.54)	7.52	(21.01)	5.03	(17.10)	*df_1_* = 2, *df_2_* = 505.9, *F* = 1.9, *p* = 0.151
Psychotherapy utilization (number of GOP)	0.37	(3.69)	0.90	(1.88)	0.54	(2.19)	*df_1_* = 2, *df_2_* = 526, *F* = 4.4, *p* = 0.013
Pharmacotherapy utilization (number of GOP)	0.56	(1.30)	0.42	(1.26)	0.62	(0.95)	*df_1_* = 2, *df_2_* = 509.6, *F* = 0.7, *p* = 0.512

*M, adjusted average; SD, standard deviation, based on hierarchical linear model with random effects for “multiple patients per therapist” and “multiple therapists per therapeutic approach”, averages of symptoms at end of therapy and costs in the year after psychotherapy were estimated given the characteristics of dropouts with quality-relevant reason (proportion of female patients = 83.9%, average age of patients = 37.9 years, number of therapy sessions = 13.39, depression at beginning of therapy (PHQ) = 1.48, anxiety at beginning of therapy (PHQ) = 1.59, stress at beginning of therapy (PHQ) = 0.92, somatization at beginning of therapy (PHQ) = 0.83, average inpatient costs in the year before therapy = 1318.24€, outpatient costs in the year before therapy = 498.76€, medication costs in the year before therapy = 295.52€, average work disability days in the year before therapy = 21.69 days, hospital stay days in the year before therapy = 7.03, utilization of psychotherapy (number of GOP) in the year before therapy = 0.17, utilization of pharmacotherapy in the year before therapy (number of GOP) = 0.55), the null-hypothesis of comparison is that all three group averages are equal*.

### Relation of cost savings and expenditures

Based on annual costs 1 year before and 1 year after outpatient psychotherapy (see Table 2), we calculated the relation of cost savings and expenditures. In our sample, regularly terminated therapies included on average 28.3 sessions which corresponds to an investment of 2322€ [ = 5 sessions · 45€ + (28.3 sessions−5) · 90€]. The sum of cost savings was 3787.30€ (331.72€ savings of inpatient costs−84.86€ outpatient costs + 57.44€ drug costs + 12.9 work disability days · 270€/day). That means that the cost savings are 63.1% (2322€/3787.30€ · 100%) higher than the investment for therapy sessions. The expenditures for psychotherapies of dropouts with unproblematic reasons were on average 16 sessions respectively 1215€ [ = 5 sessions · 45€ + (16.0 sessions−5) · 90€]. Cost savings of 1231.13€ (1096.90€ inpatient costs−7.88€ outpatient costs + 82.71€ drug costs + 0.22 work disability days · 270€/day) were 1.3% lower than the investment. Cost savings of dropouts with quality-relevant reasons were mainly driven by reduction of work disability days. The average cost savings were 2234.87€ (444.09€ inpatient costs−373.91€ outpatient costs−346.31€ drug costs + 9.3 work disability days · 270€/day) respectively 127.8% of invested sum for therapy sessions (13.4 sessions respectively 981.00€). For the entire sample, we calculated cost savings and expenditures by weighting the group specific values with the group frequency. The overall average session number was 23.6 sessions which corresponds to an investment of 1897.47€. The overall average cost saves were 3169.25€ with the largest portion caused by a reduction of work disability days (weighted average: 10.8 days). In all, for the entire sample the cost savings were 67.0% higher than the expenditures for the therapy sessions.

### Association of symptom and cost reduction

Partial correlations between symptom reduction and reduction of annual sums of health care costs are listed in Table 3. The reduction of the multiple status indicator which is the average over the PHQ scales depression, anxiety, stress, and somatization was associated with the reduction of work disability days and reduction of psychotherapy utilization. The same can be stated for the stress and somatization subscales of the PHQ. Furthermore, we found that higher reduction of anxiety was related to higher reduction in work disability days, although all of the significant correlations were low (0.85 < *r* < 0.135).

Multi-level analyses validated the correlative analyses (see Table 4). The reduction of symptoms (respectively the change of MSI) was significantly associated with reduction of work disability days and reduction of psychotherapy utilization. Moreover, we found that reduction of symptoms predicted a reduction of hospital stay days. Interestingly, there was a marginal significant relationship between a higher number of therapy sessions and a reduction of psychotherapy utilization in the year after the examined psychotherapy (standardized regression coefficient: β = 0.072, *p* < 0.1).

**Table 4:** Partial correlations between reduction of symptoms and reduction of cost variables (N = 584, *p < 0.05, **p < 0.01, two-sided tests, controlled for age and sex).

	**Inpatient costs**	**Outpatient costs**	**Drug costs**	**Work disability days**	**Hospital stay**	**Psycho-therapy (number of GOP)**	**Pharmaco-therapy (number of GOP)**
Multiple status indicator	0.017	0.030	0.038	0.098^*^	0.062	0.089^*^	0.003
depression (PHQ)	−0.002	0.021	0.005	0.049	0.045	0.062	−0.031
anxiety (PHQ)	0.027	0.010	0.062	0.085^*^	0.061	0.062	0.008
stress (PHQ)	0.022	0.036	0.023	0.091^*^	0.064	0.110^**^	0.018
somatization (PHQ)	0.011	0.061	0.028	0.135^**^	0.035	0.095^*^	0.035

## Discussion

In the present study, we examined outpatient psychotherapies regarding changes of impairment and economic aspects. We distinguished three groups: patients regularly terminating therapies (*N* = 389), patients with early termination due to an unproblematic reason (*N* = 58), and early terminators due to a quality-relevant reason (*N* = 137).

### Who terminated outpatient therapies earlier?

First, we compared the three groups with regard to socio-demographic data. Patients with regularly terminated therapies were most often male and older than patients with early terminated therapies. Furthermore, dropouts with quality-relevant reason had most often no high school graduation and most often were unemployed. These findings are in line with the meta-analysis of Swift and Greenberg (2012) and underline the validity of our results.

Interestingly, we found that patients with unproblematic dropout reason are the youngest group and had most often a high school graduation and a permanent relationship with a supportive partner. Furthermore, this patient group had the lowest proportion of severe depression (ICD10 F32.2/3, F33.2/3) and the highest proportion of adjustment disorders (F43.2). It may be assumed that young persons with high school degree have to move house more often due to a new job or career path. In such cases they have to manage a new job and possibly a long-distance relationship which can result in adjustment disorders. An additional explanation can be that patients with unproblematic dropout reason started therapy because of specifically burdening live events which is related to physical health. We observed that inpatient costs of these patients increased massively from the 2nd to the 1st quarter before outpatient psychotherapy and that these patients had the largest quarter average in the 1st quarter after psychotherapy (see Figure 2). The same can be stated for hospitalization days.

### Improvement of symptoms depending on type of therapy termination

We considered changes of symptoms from beginning to the end of therapy as well as changes of direct health care cost 1 year before vs. 1 year after outpatient psychotherapy. In concordance with our assumption, patients who regularly terminated their therapy showed the largest symptom reduction (*d* = 0.981, …, 1.22, *p* < 0.001). Additionally, we observed small but significant effects of symptom reductions for patients who terminated their therapy prematurely. Interestingly, the amount of symptom reduction was larger in the group of dropouts with quality-relevant reason than in the group of dropouts with unproblematic reason (e.g., depression: *d* = 0.429 vs. *d* = 0.366, or anxiety: *d* = 0.332 vs. *d* = 0.187, see Table 2). However, it should be noted that we considered no randomized groups. Rather, the group assignment is confounded by various selection effects (e.g., external life events like change of residence by dropouts with unproblematic reason or a misfit of patient and therapist by dropouts with quality-relevant reason). Moreover, the number of therapy sessions is different. The relative small improvement with respect to dropouts with quality-relevant reason, for example, was based on a treatment with on average 13.4 sessions whereas the large improvement of patients with regularly terminated therapies was based on 28.3 sessions. On that reasons, the pre-post effect sizes of groups are not comparable. Due to this fact, we applied a HLM and based on the regression coefficients we estimated adjusted outcome averages (see Table 3) which refer to sample characteristics of dropouts with quality-relevant reason (e.g., assuming that in all three groups a therapy with 13.4 sessions was applied). Comparisons of adjusted outcome averages revealed that patients with regularly terminated therapy had the lowest symptom impairment at end of therapy. Unproblematic dropout and quality-relevant dropout had similar adjusted averages at end of therapy which were lower than the initial symptom load. These results suggested that dropouts respond only in a sub-optimal way on outpatient psychotherapy.

The significant symptom reductions suggest that patients benefit from outpatient psychotherapy on average even in the case of early therapy termination by a problematic reason (e.g., misfit of patient and therapist or patient refused the indicated therapy). This raises the question if early therapy terminations are really failures of therapy. The literature concerning failures in psychotherapy usually distinguishes between four major events which are described as failures: patient who refuse therapy, patients who drop out from therapy, non-responders and relapsing patients (Fischer-Klepsch et al., 2009). It is doubtful that each dropout before regular ending of the therapy is a failure because patients sometimes improved systematically. Already in 1969 authors questioned the validity of the dropout = failure equation (Meyer, 1969). Results of Pekarik (1983) are in line with this. The author described the heterogeneity of the dropout group consisting of patients who drop out because they do not have a need for service, patients who drop out because of environmental constraints and patients who drop out due to problems with the service or a mismatch with the therapist. Interestingly, patients who drop out due to the two first reasons showed a symptom reduction. This is especially relevant with respect to the group of patients who terminate therapy because of environmental constraints. The authors conclude that the improvement in symptoms in this group might allow the disruption due to environmental constraints. Therefore, a premature termination of therapy does not have to be, by default, a failure of therapy (Pekarik, 1983). However, premature termination from therapy inhibits them to improve even better, to stabilize already reached successes or to learn something about the prevention of a relapse (Fischer-Klepsch et al., 2009). On the other hand, premature termination due to a misfit of the therapist-patient dyad does not have to be a failure of therapy. Maybe another treatment approach or another therapist might better help the patient. Therefore, it could be that patients who drop out of therapy are more assertive, know what they want and which treatment and therapist they need (Watson et al., 2003). Further research is definitively needed to re-define the construct of failure in psychotherapy.

### Changes in economic variables depends on the type of therapy termination

With regard to direct health care costs, we found significant reductions of annual inpatients costs for patients with regularly terminated therapies (diff = 331.72€, *p* = 0.005, see Table 2) and hospitalization days (diff = 2.55 days, *p* = 0.001). Furthermore, the number of work disability days decreased by 12.9 days (*p* < 0.001). Assuming that one work disability day means 270€ operating company expenses (sum of 105€ production loss and 165€ loss of gross value added, see (Bundesanstalt für Arbeitsschutz und Arbeitsmedizin, 2016), this means additional savings of 12.9 · 270€ = 3483€ per year. These findings are in line with Altmann et al. (2016) and Kraft et al. (2006) as well as the review of Gabbard et al. (1997). From the perspective of the patients, outpatient psychotherapy improves their mental health (Steffanowski et al., 2012; Altmann et al., 2014a,b, 2015; Strauss et al., 2015) in terms of the reduction of work disability days and hospitalization days.

For dropouts with unproblematic reason, we did not find significant changes of health care costs. Interestingly, we observed the largest reduction of inpatient costs (diff = 1096.90€, *p* = 0.392) and medication costs (diff = 82.71€, *p* = 0.189) for these patients. Outpatient costs increased marginally significant by 7.88€. We assume that the small sample size of *N* = 58 and the large variance of health care costs are reasons for non-significance. A sample size calculation showed, for example, that for a significant change of inpatient costs (diff = 1096.90€) a sample size of *N* = 144 is needed (given *r* = 0.231, α = 0.05, and power = 0.8). As mentioned above, quarter averages of inpatient costs and hospitalization days suggest that the examined outpatient therapy might be accompanied by an inpatient treatment.

Regarding dropouts with quality-relevant reason, inpatient costs, outpatient costs, and medication costs did not change significantly. However, we found a significant reduction of work disability days (diff = 9.29 days, *p* = 0.026) and hospitalization days (diff = 3.0 days, *p* = 0.032). The decrease of work disability days corresponds to savings of operating company expenses about 9.29 · 270€ = 2508.30€ per year. These findings are in a line with the symptom reductions reported above. Furthermore, we found a significant reduction of psychotherapy utilization comparing the year before and after the considered therapy. Dropouts with quality-relevant reason benefit from outpatient psychotherapy in terms of lower utilization of medical care.

Since the three sub-groups were not randomized, we estimated adjusted averages of annual costs in the year after psychotherapy (see Table 3). Assuming the characteristics of the sample with quality-relevant dropouts (e.g., highest initial impairment or smallest session number), the analysis revealed that the highest inpatient costs, work disability days and hospital stay days can be expected for dropouts with unproblematic reason. This also supports the assumption that these patients might be accompanied by an inpatient treatment or that an inpatient treatment followed the outpatient therapy. However, it should be noted that the adjusted group averages did not differ significantly regarding these cost variables. Significant differences were found for utilization of psychotherapy in the year after considered outpatient psychotherapy. Dropouts with unproblematic reasons had the highest utilization. This suggests that a relative majority of these patients continued outpatient psychotherapy. Unfortunately, we had no information about the circumstances of subsequent psychotherapy. Possibly, psychotherapy was continued with the same therapist and the discontinuation was caused by the processing time needed to process therapy extension. Alternatively, a patient might have started a new psychotherapy with another therapist. Future studies should document such facts in follow-up assessments.

Taking the relation of expenditures for therapy sessions and cost savings in terms of reduction of inpatient, outpatient, and drug costs as well as reduction of work disability days into the account, we found for each sub-group that the invested sum on average was smaller than cost savings. For the entire sample, cost savings were 67% higher than therapy costs whereby cost savings were mainly caused by the reduction of work disability days and reduction of inpatient costs. The relation was similar for patients with regularly terminated therapy (63.1%), smaller for dropouts with unproblematic reason (1.3%) and higher for dropouts with quality-relevant reason (127.8%). The findings are in line with the study of Wunsch et al. (2013), who found that the benefit of psychotherapy exceeded the therapy cost with 10 or more sessions. According to Vasiliadis et al. (2016) it can be concluded that from the perspective of society, outpatient psychotherapy is an investment which can pay off in terms of lower inpatient costs and work disability days or lower loss of gross value added.

### Do symptom and cost reduction correspond to each other?

We also examined the association between symptom reduction and reduction of direct health care costs. Against our expectation, correlations were only significant in few specific cases. The first significant finding was that the higher the reduction of anxiety, stress or somatization the higher the reduction of work disability days. This is in line with the study of Kraft et al. (2006). They reported a non-significant correlation between change of psychological distress and change of medical cost and a significant correlation between change of somatic distress and change of medical costs (*r* = 0.24, *p* = 0.002, one-sided test, *N* = 132). Furthermore, Kraft et al. (2006) reported a marginally significant correlation between change of somatic distress and change of hospitalization days (*r* = 0.11, *p* = 0.098, *N* = 132).

The second main finding is based on correlation and multi-level regression analyses. We found that higher reduction of symptoms is associated with a decrease of psychotherapy utilization in the year after the considered therapy (see Tables 4, 5). In other words: Patients with significant improvement (symptom reduction) have on average no need for further psychological treatment. Interestingly, there was a marginal significant relationship between higher number of therapy sessions and lower utilization of psychotherapy after considered therapy. The findings underline the economic benefit of outpatient psychotherapy. Concerns that there is an “over-utilization” of psychotherapy are not supported by these findings. However, our results are not in line with Fenger et al. (2014) which found an increase in therapy utilization by 296% comparing the 4th year before psychotherapy with the 4th year after psychotherapy. The increase with respect to the control group was only 99%. Also, Lazar et al. (2006) reported an increase of psychiatric health care utilization both after psychotherapy and after psychoanalysis. Maljanen et al. (2016) compared cost-effectiveness of short-term and long-term psychotherapy in the treatment of affective and anxiety disorders. Due to the lower therapy costs of short-term therapies, they were more cost-effective than long-term therapy. However, these cost-effectiveness analyses examined incremental cost-effectiveness ratios whereas the presented study considered a large body of direct costs provided by health insurant funds. In concordance to our results, Abbass et al. (2015) found a significant reduction of hospital costs for patients treated with intensive short-term PDT whereas the untreated control group showed higher values than the baseline in the first and second post-treatment year. Currently, the number of studies is low and methodology is heterogeneous (e.g., regarding considered time intervals, patient characteristics, treatment, and statistical methods) so that in our opinion no final conclusion can be drawn. Furthermore, it should be examined under which conditions symptoms decrease but cost increase and under which conditions symptoms and costs decrease simultaneously.

Table 5Regression analysis using hierarchical linear models.

**Table:** 

	**Change of inpatient costs**			**Change of outpatient costs**			**Change of drug costs**		
	***b***	***SE***	**β**	***b***	***SE***	**β**	***b***	***SE***	**β**
Intercept	272.5	(627.2)	0.018	152.9	(339.7)	0.202	−13.7	(400.9)	0.000
Gender (female = 1)	147.2	(323.6)	0.012	98.7	(91.8)	0.035	46.7	(208.7)	0.009
Age	3.3	(10.8)	0.009	11.8^***^	(3.1)	0.129	13.8^*^	(7.0)	0.079
Inpatient cost year before	−1.0^***^	(0.1)	−0.787	0.0	(0.0)	0.042	−0.1^+^	(0.1)	−0.153
Outpatient cost year before	−0.1	(0.2)	−0.009	−0.9^***^	(0.0)	−0.650	0.0	(0.1)	−0.006
Drug costs year before	0.0	(0.1)	0.008	0.0	(0.0)	0.029	−0.5^***^	(0.1)	−0.353
Work disability days before	8.5^**^	(2.8)	0.088	−0.4	(0.8)	−0.019	−0.3	(1.8)	−0.006
Hospital stay days before	3.2	(14.2)	0.014	−1.6	(4.0)	−0.030	19.2^*^	(9.2)	0.185
Psychotherapy util. before	−398.4	(273.1)	−0.041	185.6^*^	(77.6)	0.082	−22.1	(177.6)	−0.005
Pharmacotherapy util. before	166.4	(139.5)	0.033	61.1	(39.6)	0.052	9.0	(90.5)	0.004
Number of sessions of current PT	−6.5	(6.7)	−0.027	−0.5	(1.9)	−0.009	−4.8	(4.3)	−0.045
Multiple status indicator baseline	449.2	(346.6)	0.040	197.5^*^	(98.7)	0.075	−178.0	(225.4)	−0.036
Multiple status indicator change	498.3	(340.8)	0.046	125.2	(97.6)	0.048	24.3	(221.3)	0.005

**Table:** 

	**Change of work disability days**			**Change of hospital stay days**			**Change of psychotherapy utilization**			**Change of pharmacotherapy utilization**		
	***b***	***SE***	β	***b***	***SE***	β	***b***	***SE***	β	***b***	***SE***	β
Intercept	0.4	(6.1)	0.012	2.9	(2.8)	0.034	−0.1	(0.2)	0.062	0.1	(0.2)	0.007
Gender (female = 1)	−0.2	(3.1)	−0.001	0.7	(1.4)	0.011	0.0	(0.1)	0.004	−0.1	(0.1)	−0.047
Age	0.0	(0.1)	−0.001	0.0	(0.0)	−0.007	0.0	(0.0)	0.041	0.0	(0.0)	0.045
Inpatient cost year before	0.0	(0.0)	−0.010	0.0^+^	(0.0)	−0.100	0.0	(0.0)	0.030	0.0	(0.0)	0.026
Outpatient cost year before	0.0	(0.0)	−0.008	0.0	(0.0)	−0.005	0.0	(0.0)	−0.004	0.0	(0.0)	−0.013
Drug costs year before	0.0	(0.0)	−0.021	0.0	(0.0)	0.017	0.0	(0.0)	−0.031	0.0	(0.0)	−0.021
Work disability days before	−0.9^***^	(0.0)	−0.812	0.0^***^	(0.0)	0.088	0.0	(0.0)	−0.013	0.0	(0.0)	−0.064
Hospital stay days before	−0.1	(0.1)	−0.032	−0.9^***^	(0.1)	−0.734	0.0	(0.0)	−0.015	0.0	(0.0)	0.046
Psychotherapy util. before	1.1	(2.6)	0.011	−3.0^*^	(1.2)	−0.062	−0.8^***^	(0.1)	−0.410	0.1	(0.1)	0.053
Pharmacotherapy util. before	0.6	(1.3)	0.010	−0.1	(0.6)	−0.004	0.0	(0.0)	0.025	−0.4^***^	(0.0)	−0.420
Number of sessions of current PT	−0.1	(0.1)	−0.033	0.0	(0.0)	−0.012	0.0^+^	(0.0)	0.072	0.0	(0.0)	−0.047
Multiple status indicator baseline	12.9^***^	(3.3)	0.106	2.1	(1.5)	0.037	0.4^***^	(0.1)	0.168	0.1	(0.1)	0.049
Multiple status indicator change	7.6^*^	(3.2)	0.064	3.8^*^	(1.5)	0.069	0.3^**^	(0.1)	0.148	0.1	(0.1)	0.026

*b, regression coefficient; SE, standard error; β, standardized coefficient*,

*** *p < 0.001*,

** *p < 0.01*,

* *p < 0.05*,

+ *p < 0.1, sample size was N = 584, dependent variables were the change of costs respectively annual sum of the year after current outpatient psychotherapy minus annual sum of the year before current therapy, due to space limitations random effects not reported*.

The third finding was that outpatient costs, inpatient costs, and medication costs were not significantly correlated with the change of symptoms from pre- to post-psychotherapy. However, multi-level analysis revealed an association between symptom reduction (assessed as pre-post-change of MSI) and reduction of hospital stay days. The findings are only in part in line with the study of Kraft et al. (2006) which did not found an association between change of psychological or somatic distress on the one side and change of medical costs and hospitalization days on the other side. The non-confirmed relationship between symptom reduction and inpatient costs is remarkable because we found significant reductions of inpatient costs and significant reductions of symptoms for patients who regularly terminated psychotherapies so that an association between change of symptoms and change of inpatients costs could be assumed. An explanation can be that outpatient psychotherapy can be a part of an ambulatory medical aftercare or can be embedded in a somatic inpatient treatment or other physical treatments. In such cases, psychotherapy has mainly a supporting function and is not the reason for the observed reduction of inpatient costs or hospitalization days. Another explanation that cost reduction and symptom reduction were uncorrelated can be that cost data have a skewed distribution and that cost data have a large variance in comparison to questionnaire data. Accordingly, large samples are needed to get robust estimates of averages (small standard errors) and to analyze marginal conditions of cost reduction (e.g., a simultaneous ambulatory medical aftercare). However, our study is the second about the correlation of cost reduction and symptom reduction. Future research is needed on this topic.

### Strengths and limitations

Since all patients participated voluntarily, the sample might not have been representative for psychotherapy patients in Germany. Additionally, due to the limited period of data collection, shorter therapies which might represent less complicated and thus relatively effective therapy trajectories were overrepresented in the sample. This could have caused an overestimation of cost reduction due to psychotherapy. However, it should be noted that our analysis included regularly and prematurely terminated psychotherapies. We also included cost data of dropouts. Furthermore, the amount of changes of inpatient costs, work disability days, and hospitalization days of patients with regularly terminated therapies are very similar to the amount reported by Altmann et al. (2016) which examined health care cost reductions of 22,294 patients and similar to the amount reported in the review of de Maat et al. (2007). This underlines the validity of our results.

Furthermore, due to the project aim to evaluate the feasibility and the acceptance of the therapy documentation, no control group without psychotherapy was included to compare post-pre-differences of the treatment group. In other studies, control groups showed an overall cost increase between pre and post psychotherapy of an average of 12.3% (Chiles et al., 1999) whereas we found mostly non-significant changes or a decrease of inpatient cost and work disability days.

A further limitation is that the considered time interval for cost data was limited to 1 year before respectively 1 year after psychotherapy. Studies show a further change of costs during 2 years before respectively 2 year after psychotherapy (Altmann et al., 2016). In this study, we had data of the second year before and after outpatient therapy, but the proportion of missing values was too high to be meaningfully imputed. Therefore, we restricted our analysis to 1 year before and after outpatient therapy. Planning future studies the amount of time and money needed for a long-term study including follow-up (many outpatient therapies in Germany last more than 1 year) as well as issues of data management (e.g., a delay between treatment and deliver of related cost data by health insurance fund can take up 9 months) should be taken into the account (Strauss et al., 2015).

A critical point is that missing questionnaire data and cost data can be imputed simultaneously using the missForest imputation. We decided against this because missing questionnaire data occurred not at random: only if a therapy terminated prematurely, then the post treatment measure is not assessed (see above). We had no dropout patient with a valid post measure. We tested the result of imputation, if this fact is ignored. As result the impairment at end of therapy of dropouts is similar to patients with regular therapy termination which is not in concordance with the literature. On that reason, we imputed missing post treatment values of dropout with the conservative method last observation carried forward and imputed in a second step cost data with missForest imputation. Future studies should have formal conditions that patients can be contacted for follow-up assessments even in the case of prematurely therapy termination so that such difficult data situation can be avoided.

It can also be seen as critical that the sample is disorder-heterogeneous and that the treatment is heterogeneous (cognitive behavioral, psychodynamic, and psychoanalytic therapy), too. However, the study aim was not to evaluate the effect of a specific treatment on a specific disorder. Moreover, the study aim was to map psychotherapy under naturalistic condition with all their diversity of patients and therapists.

Finally, using costs as a measure for utilization of health care services can be questioned, since costs are highly dependent on price negotiations with health insurances and on the pricing policy of pharmaceutical companies. Both interrelates with the economic framework and is constantly developing. On the other hand, also billing details determine the prices: If for example fixed rates are accounted for, the same price will apply to one or 10 visits to the doctor.

Studies as comprehensive as this one, covering various health insurances' clients as well as different health care domains (outpatient and inpatient care) are seldom reported. Although, the use of objective data from different sources clearly provides a variety of problems with data handling that should be considered in planning naturalistic treatment studies (Strauss et al., 2015).

It is important to note that psychotherapy aims at reducing psychological symptoms and enhancing the patient's quality of life and subjective well-being. Whether the treatment is economical, adequate, necessary and convenient, as stipulated by the German Social Law, cannot be proven by the observation of long-term cost development. Still, a proven long-term cost-effectiveness of psychotherapy might serve as an economic incentive for health insurances to optimize health care toward a guaranteed supply of and access to indicated psychotherapy.

## Conclusions

The findings of symptom and cost reduction for patients with regularly terminated therapy and patients with a quality relevant dropout suggested that outpatient psychotherapy is effective to treat mental disorders under naturalistic conditions, even in terms of health economics evaluation. It seems that not each dropout is a therapy failure, especially since we observed a large decrease of work disability days within both groups.

## Ethics statement

Ethics Commission: Jena University Hospital. Planning of the QS-PSY-BAY project, sampling, recruitment, and data assessment have been performed by our ordering customer the Bavarian Administration of Statutory Health Care Physicians (KVB). The Otto-Selz-Institute of Applied Psychology (University of Mannheim) was a subcontractor of KVB and performed the assessment of questionnaire data. All patients which were included in the questionnaire study provided a written informed consent. Participation was voluntary and anonymous. Furthermore, the German data protection standards were applied and certified by the German Federal Ministry for Information Security. Data were encrypted and anonymized, data storage and transfer were protected against unauthorized data access. After assessment ordering customer or subcontractors of KVB provided us (Jena University Hospital, Institute of Psychosocial Medicine and Psychotherapy) anonymous datasets. The Jena University Hospital was a subcontractor of KVB and responsible for data matching using an anonymous identification number and data analyses. The KVB declared that the project fulfilled the legal laws of data protection and data safety and the Ethical Guidelines of the WHO in all parts, such as the Declaration of Helsinki. Additionally, the ethical aspects of the presented data analyses were proofed by the Ethics Commission of Jena University Hospital.

## Author contributions

The research question for these analyses was designed by UA. EB and IP represent the group of psychotherapist who initiated the QS-PSY-BAY project. AF represents the Bavarian Administration of Statutory Health Care Physicians which coordinated the QS-PSY-BAY project. AS represents the working group that assessed the questionnaire data. UA, AZ, and DT prepared the data for analysis (e.g., matching of questionnaire and cost data or missing data imputation). UA and AZ analyzed the data. The discussion of results was performed by all authors. UA, AZ, and DT drafted the manuscript whereby UA was primary responsible. All authors read the manuscript and gave critical comments. All authors approved the final version of the manuscript.

### Conflict of interest statement

The authors declare that this study received funding from Association of Health Insurance Companies (in German: Verband der Ersatzkassen). The funder was not involved in the study design or collection, analysis, or interpretation of the data.
